# 不同转移部位的非小细胞肺癌患者的生存时间比较

**DOI:** 10.3779/j.issn.1009-3419.2019.02.05

**Published:** 2019-02-20

**Authors:** 炳群 吴, 慎海 魏, 进涛 田, 小平 宋, 鹏程 胡, 永 崔

**Affiliations:** 1 100016 北京，清华大学第一附属医院胸外科 Department of Thoracic Surgery, First Affiliated Hospital of Tsinghua University, Beijing 100016, China; 2 100050 北京，首都医科大学附属北京友谊医院胸外科 Department of Thoracic Surgery, Beijing Friendship Hospital Affiliated to Capital Medical University, Beijing 100050, China

**Keywords:** 肺肿瘤, 转移, 中位生存时间, Lung neoplasms, Metastasis, Median survival time

## Abstract

**背景与目的:**

肺癌是全球范围内发病率和死亡率最高的恶性肿瘤，导致其高病死率的主要原因是局部复发和远处转移，而转移部位对患者的预后有一定预测作用，本研究旨在比较不同远处转移部位的非小细胞肺癌（non-small cell lung cancer, NSCLC）患者的生存时间。

**方法:**

从美国监测、流行病学和最终结果（Surveillance, Epidemiology, and End Result, SEER）数据库中提取出2010年-2014年间确诊的NSCLC患者共117, 542例，回顾性分析其远处转移部位与生存时间的关系。

**结果:**

在确诊的117, 542例NSCLC患者中，42, 071例（35.8%）患者病史中发生不同程度的远处转移，其中单器官转移26, 932例，多器官转移15, 139例，分别占转移患者总数的64.0%和36.0%。病史中无转移的患者的中位生存时间是21个月；而在病史中有转移患者中，中位生存时间分别是7个月（肺）、6个月（脑）、5个月（骨）、4个月（肝）、3个月（多器官转移），差异显著（*P* < 0.001，肝转移与多器官转移除外*P*=0.650）；且绝大多数NSCLC患者（88.4%）最终死于肺癌。

**结论:**

NSCLC患者远处转移提示预后差，在单器官转移患者中，肺转移预后最佳，肝转移预后最差；多器官转移患者预后差于单器官转移。

## Abstract (en)

**Background and objective:**

The purpose of this study is to compare the survival time of non-small cell lung cancer (NSCLC) patients with different organ metastasis. Among all cancers, the morbidity and mortality of lung cancer is the highest worldwide, which may caused by local recurrence and distant metastasis, and the location of metastasis may predict the prognosis of patients.

**Methods:**

A total of 117, 542 patients with NSCLC diagnosed between 2010 and 2014 were enrolled from Surveillance, Epidemiology, and End Result (SEER) databases, and the relationship between distant metastasis and survival time was retrospectively analyzed.

**Results:**

Of all the 117, 542 patients diagnosed with non-small cell lung cancer, 42, 071 (35.8%) patients had different degrees of distant metastasis during their medical history, including 26, 932 single organ metastases and 15, 139 multiple organ metastases, accounting for 64.0% and 36.0% of the metastatic patients respectively. Compared with patients with no metastasis, whose median survival time was 21 months, the median survival time of patients with metastases was 7 months (lung), 6 months (brain), 5 months (bone), 4 months (liver), and 3 months (multiple organ) respectively, and the difference was significant (*P* < 0.001, except liver vs multiple organ *P*=0.650); Most patients with NSCLC (88.4%) eventually died of lung cancer.

**Conclusion:**

Distant metastasis of NSCLC patients indicates poor prognosis. In NSCLC patients with single organ metastasis, the prognosis of lung metastasis is the best, and liver metastasis is the worst, and multiple organ metastasis is worse than single organ metastasis.

肺癌是全球范围内发病率和死亡率最高的恶性肿瘤，非小细胞肺癌（non-small cell lung cancer, NSCLC）约占肺癌的80%^[1]^。同样，在我国，肺癌的发病率、死亡率位于第一位，导致其高病死率的主要原因是局部复发和远处转移^[2]^，而肺、脑、骨、肝等是肺癌的常见转移部位^[3]^。

## 1 对象和方法

### 1.1 对象

本研究直接从美国监测、流行病学和最终结果（Surveillance, Epidemiology, and End Result, SEER）数据库中筛选数据，该数据库覆盖美国28%的人群。我们筛选数据时使用的软件是SEER^*^Stat 8.3.4版，本研究通过我院伦理委员会评审。

#### 1.1.1 筛选标准

2010年-2014年病理确诊的NSCLC患者，排除既往患有恶性肿瘤的患者，最终选出117, 542例。我们从数据库中提取出了每位患者的年龄、性别、种族、婚姻状况、病理、分化程度、手术、T分期及N分期[病理分期，基于美国癌症联合会（American Joint Committee on Cancer, AJCC）第6版分期]、生存时间、放疗、化疗、肿瘤转移部位等。

#### 1.1.2 分组

我们将提取出的NSCLC患者分为两组，将在2010年-2014年间未发生转移的NSCLC患者分为无转移组，将2010年-2014年确诊NSCLC时已存在远处转移或确诊NSCLC后发生远处转移的患者分为转移组。我们将转移组进一步分为脑转移、骨转移、肝转移、肺转移及多器官转移。在进行数据分析时，我们将年龄分为4组（≤60岁、61岁-70岁、71岁-80岁以及 > 80岁），把手术方式分为6组（未手术，亚肺叶、肺叶、全肺、手术方式未知、未知是否手术），其中亚肺叶组包括肿瘤的局部破坏（例如局部伽马刀治疗、射频消融治疗）、局部切除以及楔形切除、肺段切除。肺叶切除包括单肺叶切除和双肺叶切除。放疗及化疗不特指术前或术后放化疗，病史中曾进行过放疗或化疗，即认为该患者进行了放疗或化疗。

### 1.2 统计学处理

用SPSS 22.0统计分析软件，应用*Kaplan-Meier*法分析绘制生存曲线，差异性检验使用*Log-rank*检验，*P* < 0.05为差异有统计学意义。

## 2 结果

### 2.1 基本特点

我们从SEER数据库中共筛选出的117, 542例患者，其基本临床特点详见表 1。起始时间是2010年1月，截止时间是2014年12月，随访期间内，72, 699例（61.8%）患者死亡，总体中位生存时间是12个月；在所有死亡患者中，64, 292例（88.4%）死于肺癌，只有8, 407例（11.6%）死于其他疾病；42, 071例（35.8%）患者发生远处转移，是否发生远处转移与患者年龄、病理、T分期、N分期、分化程度、手术方式、放疗、化疗关系密切（*P* < 0.050）：随着年龄增大，肺癌患者发生远处转移的几率逐渐减小（在年龄≤60岁组，转移率为43.3%，而在年龄 > 80岁组，降低到30.6%）。不同病理类型患者，病史中发生远处转移的几率不同，腺癌患者明显高于鳞癌患者（41.1% *vs* 25.0%）。分化程度越差，远处转移发生的可能性越大（分化好15.3% *vs*未分化34.3%）。在T及N分期中，分期越晚，远处转移的几率越大（T0除外）。在手术组，手术患者发生远处转移的概率低（亚肺叶、肺叶、全肺分别为11.6%、2.2%、3.2%）。而在进行放疗和化疗患者组中，远处转移几率高。

**1:** 纳入患者的临床特征 Clinical characteristics of the patients selected

Clinical characteristics	*n* (%)	Metastasis (%)					*P*
		Single organ				Multiple organ	
		Brain	Bone	Liver	Lung		
Gender							< 0.001
Male	35, 923 (65.5%)	3, 151 (5.7%)	3, 854 (7.0%)	1, 096 (2.0%)	4, 060 (7.4%)	6, 803 (12.4%)	
Female	39, 548 (63.1%)	3, 390 (5.4%)	5, 626 (9.0%)	1, 410 (2.2%)	4, 360 (7.0%)	8, 336 (13.3%)	
Age (yr)							< 0.001
≤60	16, 232 (56.7%)	2, 457 (8.6%)	2, 599 (9.1%)	555 (1.9%)	1, 784 (6.2%)	5, 016 (17.5%)	
61-70	24, 380 (64.3%)	2, 216 (5.8%)	3, 017 (8.0%)	812 (2.1%)	2, 430 (6.4%)	5, 046 (13.3%)	
71-80	23, 544 (67.9%)	1, 415 (4.1%)	2, 650 (7.6%)	713 (2.1%)	2, 651 (7.6%)	3, 715 (10.7%)	
> 80	11, 315 (69.4%)	453 (2.8%)	1, 213 (7.4%)	426 (2.6%)	1, 541 (9.4%)	1, 362 (8.4%)	
Race							< 0.001
White	60, 481 (65.0%)	5, 107 (5.5%)	7, 535 (8.1%)	2, 017 (2.2%)	6, 439 (6.9%)	11, 478 (12.3%)	
Black	8, 982 (62.2%)	888 (6.2%)	1, 149 (8.0%)	330 (2.3%)	1, 152 (8.0%)	1, 928 (13.4%)	
Others	6, 008 (59.7%)	546 (5.4%)	795 (7.9%)	159 (1.6%)	815 (8.1%)	1, 733 (17.2%)	
Histology							< 0.001
Squamous	27, 652 (75.0%)	1, 093 (3.0%)	2, 173 (5.9%)	846 (2.3%)	2, 475 (6.7%)	2, 645 (7.2%)	
Adenocarcinoma	43, 882 (58.9%)	5, 011 (6.7%)	6, 846 (9.2%)	1, 469 (2.0%)	5, 608 (7.5%)	11, 656 (15.7%)	
Others^*^	3, 937 (63.6%)	437 (7.1%)	460 (7.4%)	191 (3.1%)	323 (5.2%)	838 (13.5%)	
Differentiation							< 0.001
Well	6, 237 (84.7%)	113 (1.5%)	203 (2.8%)	33 (0.4%)	467 (6.3%)	309 (4.2%)	
Moderate	20, 141 (79.4%)	818 (3.2%)	1, 165 (4.6%)	252 (1.0%)	1, 321 (5.2%)	1, 672 (6.6%)	
Poor	22, 266 (67.5%)	2, 021 (6.1%)	2, 246 (6.8%)	636 (1.9%)	2, 140 (6.5%)	3, 694 (11.2%)	
Undifferentiated	796 (65.7%)	92 (7.6%)	101 (8.3%)	16 (1.3%)	72 (5.9%)	135 (11.1%)	
Unknown	26, 031 (51.4%)	3, 497 (6.9%)	5, 764 (11.4%)	1, 569 (3.1%)	4, 406 (8.7%)	9, 329 (18.4%)	
T-stage							< 0.001
T0	258 (46.1%)	100 (17.9%)	101 (18.0%)	28 (5.0%)	10 (1.8%)	63 (11.3%)	
T1	19, 588 (83.6%)	945 (4.0%)	1, 209 (5.2%)	253 (1.1%)	385 (1.6%)	1, 051 (4.5%)	
T2	25, 598 (71.9%)	2, 407 (6.8%)	2, 553 (7.2%)	705 (2.0%)	1, 338 (3.8%)	3, 016 (8.5%)	
T3	4, 958 (73.1%)	307 (4.4%)	611 (8.8%)	107 (1.5%)	334 (4.8%)	641 (9.2%)	
T4	19, 650 (47.8%)	2, 000 (4.9%)	3, 744 (9.1%)	1, 016 (2.5%)	5, 786 (14.1%)	8, 918 (21.7%)	
Tx	5, 419 (54.9%)	782 (7.9%)	1, 261 (12.8%)	397 (4.0%)	553 (5.6%)	1, 450 (14.7%)	
N-stage							< 0.001
N0	37, 732 (80.1%)	1, 782 (3.8%)	2, 326 (4.9%)	623 (1.3%)	2, 167 (4.6%)	2, 485 (5.3%)	
N1	6, 822 (67.9%)	607 (6.0%)	853 (8.5%)	191 (1.9%)	523 (5.2%)	1, 056 (10.5%)	
N2	20, 732 (52.9%)	2, 834 (7.2%)	4, 083 (10.4%)	1, 109 (2.8%)	3, 330 (8.5%)	7, 075 (18.1%)	
N3	6, 301(43.1%)	973 (6.7%)	1, 506 (10.3%)	336 (2.3%)	1, 862 (12.7%)	3, 650 (25.0%)	
Nx	3, 884 (59.0%)	345 (5.2%)	711 (10.8%)	247 (3.8%)	524 (8.0%)	873 (13.3%)	
Surgical procedure							< 0.001
No	46, 390 (53.3%)	6, 154 (7.1%)	9, 268 (10.6%)	2, 436 (2.8%)	7, 943 (9.1%)	14, 905 (17.1%)	
Sublobectomy	5, 241 (88.4%)	109 (1.8%)	97 (1.6%)	37 (0.6%)	294 (5.0%)	152 (2.6%)	
Lobectomy	21, 912 (97.8%)	222 (1.0%)	79 (0.4%)	21 (0.1%)	126 (0.6%)	35 (0.2%)	
Pneumonectomy	1, 391 (96.8%)	17 (1.2%)	11 (0.8%)	2 (0.1%)	13 (0.9%)	3 (0.2%)	
Surgery, NOS	149 (67.1%)	28 (12.6%)	8 (3.6%)	1 (0.5%)	13 (5.9%)	23 (10.4%)	
Unknown	388 (84.0%)	11 (2.4%)	16 (3.5%)	9 (1.9%)	17 (3.7%)	21 (4.5%)	
Chemotherapy							< 0.001
Yes	26, 738 (56.6%)	5, 120 (10.8%)	4, 824 (10.2%)	405 (0.9%)	1, 776 (3.8%)	8, 375 (17.7%)	
Others^**^	48, 733 (69.3%)	1, 421 (2.0%)	4, 655 (6.6%)	2, 101 (3.0%)	6, 630 (9.4%)	6, 764 (9.6%)	
Chemotherapy							< 0.001
Yes	30, 424 (57.4%)	3, 556 (6.7%)	5, 219 (9.8%)	1, 201 (2.3%)	4, 397 (8.3%)	8, 229 (15.5%)	
Others^***^	45, 047 (69.8%)	2, 985 (4.6%)	4, 260 (6.6%)	1, 305 (2.0%)	4, 009 (6.2%)	6, 910 (10.7%)	
All	75, 471 (64.2%)	6, 541 (5.6%)	9, 479 (8.1%)	2, 506 (2.1%)	8, 406 (7.2%)	15, 139 (12.9%)	
^*^: adenosqumous carcinoma, large cell carcinoma; ^**^: no radiation or unclear; ^***^: no chemotherapy or unclear.							

### 2.2 生存分析

如表 2所示，病史中无远处转移的患者的中位生存时间是21个月，远高于有远处转移的患者的中位生存时间。在肺癌远处转移患者中，肺转移患者的中位生存时间最佳，达到7个月，脑转移患者的中位生存时间次之，为6个月，骨转移患者的中位生存时间为5个月，肝转移及多器官转移患者的中位生存时间最差，分别为4个月和3个月。

**2:** 多发转移病灶NSCLC患者的中位生存时间 The median survival time of the NSCLC patients with different metastatic sites

Metastatic sites	Median survival time (month)	95%CI	
		Lower bound	Upper bound
No	21.00	20.63	21.37
Brain	6.00	5.73	6.27
Bone	5.00	4.81	5.18
Liver	4.00	3.61	4.39
Lung	7.00	6.67	7.33
Multi-organ	3.00	2.89	3.11

从图 1中也可看出，肺癌患者的预后由好至差依次是无转移、肺转移、脑转移、骨转移、肝转移、多器官转移。除肝转移患者和多器官转移患者的生存曲线区分不明显（*P*=0.650）外，余各生存曲线之间的差别统计学意义明显（*P* < 0.001）。

**1:**
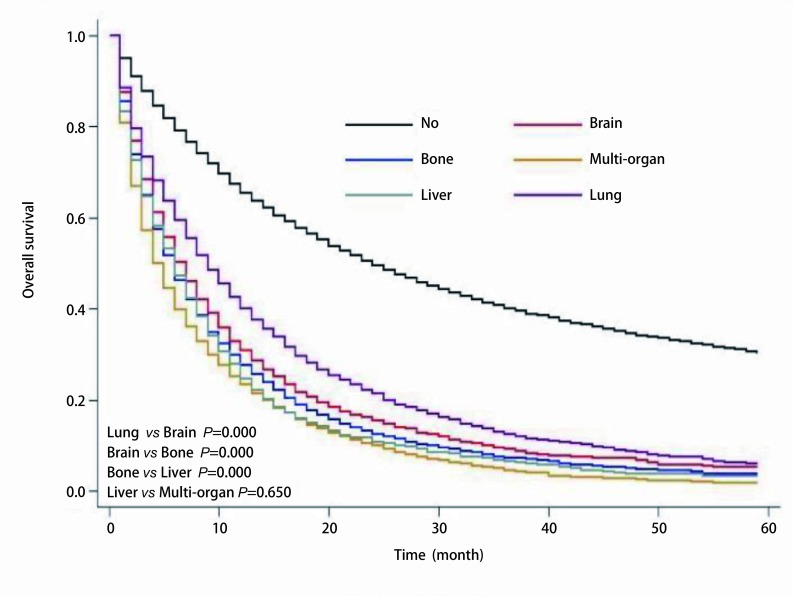
不同转移部位NSCLC患者的生存曲线（*Kaplan-Meier*法） *Kaplan-Meier* analysis for the survival curves of the NSCLC patients with different metastatic sites

## 3 讨论

近几年，随着医疗技术的进步，肺癌的治疗得到迅猛发展，NSCLC患者，尤其是腺癌患者总体生存均显著延长。但是，大多数肺癌患者初诊时即为晚期，出现不同部位的转移，其远处转移过程复杂，从癌细胞离开原发灶、侵入血液和淋巴系统，直至肿瘤细胞在远处生长，每个环节均与其生物学特性关系密切^[4]^。晚期NSCLC患者转移模式有单病灶、单器官、多病灶和多器官等多种，本研究中，我们主要比较患者病史中出现肺转移、脑转移、骨转移、肝转移以及多器官转移的生存时间。

在肺癌患者整个病史中，无远处转移的患者，中位生存期达到21（20.63-21.37）个月，明显高于有远处转移患者。在发生远处转移的患者中，肺转移患者的中位生存期最长，达7（6.67-7.33）个月，其预后好，可能与人的呼吸储备功能大有关。脑转移患者的中位生存期为6（5.73-6.27）个月，与我们的研究结果类似，Ali等^[5]^发现肺癌脑转移患者的中位生存时间是7.8个月。既往研究发现^[6-8]^，影响脑转移患者生存的因素有年龄、体能状态、转移间隔时间、转移数目、治疗方法、治疗周期、脑转移症状、颅外转移、脑转移次序、基因突变、程序性死亡受体-1等；脑转移患者的预后好于骨转移，这可能与近些年对于脑转移治疗的方式进展迅速，治疗效果越来越好。骨转移患者的中位生存期为5（4.81-5.18）个月，其生存受多种因素的影响，单发骨转移还是多发骨转移，是否合并病理性骨折，以及是否合并其他部位转移。Rief等^[9]^研究发现，单发骨转移患者6个月和12个月的OS分别是76.7%和47.2%，其OS明显高于其他部位转移的患者60.0%和34.0%；多发骨转移患者及合并病理性骨折患者的OS较差。肝转移在肺癌单器官转移中预后最差，其中位生存期仅4（3.61-4.39）个月，这与Ren^[10]^的研究内容及结果类似，他们通过对美国SEER数据库中数据分析，发现在腺癌和小细胞肺癌患者中，肝转移患者相对于其他单器官转移的患者，预后最差。不同的是我们的数据构成不同，但是同样因为在我们的数据中，腺癌患者占多数（58.1%）。多器官转移的患者预后最差，中位生存期为3（2.89-3.11）个月，类似既往研究资料^[11]^，NSCLC远处转移以多器官远处转移为主（64.0% *vs* 36.0%），而多器官转移患者，一般其肿瘤负荷高于单器官转移，其预后相对较差^[12, 13]^。如图 1所示，除肝转移组与多器官组两组间差异无统计学意义（*P*=0.650）外，余各组间差异显著（*P* < 0.001）。

如上所述，远处转移提示肺癌患者预后差，其发生的影响因素众多。从表 1中可以看出，年龄与远处转移发生概率成反比，这可能原因是低龄患者体质好，对各种治疗耐受好，总生存时间长，病史中发生远处转移的概率增加。腺癌患者远处转移发生率高于鳞癌患者，且其多器官转移率明显高于鳞癌患者（15.7% *vs* 7.2%）。其原因主要从以下几方面考虑：首先是腺癌生物学特性决定其远处转移率高于鳞癌，其次是近些年针对肺腺癌的治疗发展迅速，肺腺癌患者预后相对较好，再次是腺癌患者发病率升高。同于既往临床经验，分化程度越差、T分期及N分期越差，患者易发生远处转移。

由于NSCLC远处转移预后差，影响因素众多，近些年对不同器官转移治疗方面的研究进展迅速，预后逐渐好转。针对肺癌脑转移，化疗效果不佳，目前其治疗有手术、放疗^[14]^、免疫治疗、靶向治疗^[15]^，以及联合治疗^[16]^。对于肝转移的NSCLC患者，Vokes等^[17]^研究发现，抗程序性死亡-1抗体Nivolumab与多西他赛比，在肝转移的NSCLC患者中提高患者总生存；Ishige等^[18]^提出：在出现肝脏寡复发NSCLC患者的多学科治疗中，肝切除术也许同样有效。骨转移与骨骼相关事件显著增加有关，包括严重骨痛、高钙血症、病理性骨折、脊髓压迫症^[19]^。目前临床有阿片类药物止痛治疗；唑来膦酸和伊班膦酸^[20]^，部分患者采用放疗，小部分患者采用手术治疗^[21]^，Willeumier等^[22]^研究发现，*EGFR*突变阳性患者中位生存期可达到17.3个月（95%CI: 12.7-22.0）。肺转移患者，如原发灶与孤立性转移灶不在同一肺叶并且可手术切除，可从手术获益^[23]^。另外，转移灶的立体放疗，效果也很好^[24]^。

本研究存在以下几方面的缺陷。首先是由于SEER数据库未明确记录患者转移后的生存时间，只能概述病史中出现远处转移患者总体生存时间，且由于数据库不能提供更长时间的此类患者的信息，导致筛选的时间过短，遗漏部分患者信息，造成误差。另外，出现远处转移的时机不同，患者的总生存也会不同。比如，确诊肺癌时即发现转移，还是在治疗后期发现转移，这两种情况，患者的总生存时间可能会不同。其次，肺癌远处转移的部位很多，除了本研究中的4种常见转移部位，肾上腺转移也是其常见转移部位之一，SEER数据库中无此数据。另外，对于同样出现一个器官转移的患者，单发转移还是多发转移，未明确记录，影响预后判断。肺癌患者还有其他少见的转移部位，比如皮肤转移、脾转移等，而SEER数据库中未包含上述转移的数据。再者，从近几年的研究数据看，肺癌患者的基因突变种类、是否进行靶向治疗、免疫治疗等对其预后影响显著，这些数据我们目前无法获得。最后，由于SEER数据库2010年前的数据中，未记录远处转移情况，所以，本研究中选取的时间段较短，不能更确切地反映患者的真实生存时间。

总之，NSCLC患者病史中出现远处转移，对预后影响大，且不同转移部位，预后存在显著差异。对于确诊NSCLC的患者，尽早进行全身情况评估，明确分期，有利于更准确地判断其预后。
